# Association between self-reported hearing loss and low socioeconomic status in Japan: findings from the Toyama dementia survey

**DOI:** 10.1186/s12877-020-01680-y

**Published:** 2020-08-05

**Authors:** Nobue Nakahori, Michikazu Sekine, Masaaki Yamada, Takashi Tatsuse, Hideki Kido, Michio Suzuki

**Affiliations:** 1 Faculty of Nursing Science, Tsuruga Nursing University, 78-2-1 Kizaki, Tsuruga, Fukui, 914-0814 Japan; 2 Department of Epidemiology and Health Policy, School of Medicine, University of Toyama, 2630 Sugitani, Toyama, Toyama 930-0194 Japan; 3 Kiseikai, Kido Clinic, 244 Honoki, Imizu, Toyama, 934-0053 Japan; 4 Department of Neuropsychiatry, School of Medicine, University of Toyama, 2630 Sugitani, Toyama, Toyama 930-0194 Japan

**Keywords:** Older adults, Hearing loss, Socioeconomic status, Educational attainment

## Abstract

**Background:**

Age-related hearing loss reduces the quality of life in older adults. Low socioeconomic status (SES) has been reported as a risk factor for hearing loss, although this has not been verified in Japan. This study aimed to assess the association between low SES and hearing loss, excluding people with dementia, in Japan.

**Methods:**

Data from the Toyama Dementia Survey, Japan, were used. Overall, 126 patients with hearing loss and 913 unimpaired controls were identified. Participants’ presentation of dementia, self-reported hearing loss, history of medically diagnosed disease (hypertension, hyperlipidemia, diabetes, stroke, or angina pectoris/cardiovascular disease), lifestyle factors (alcohol consumption and smoking), and SES (educational attainment and occupation) were assessed. Any association between low SES and hearing loss was investigated using logistic regression analysis.

**Results:**

The odds ratio (OR) for hearing loss was higher for participants with low educational attainment than for those with high educational attainment (age- and sex-adjusted OR 3.08; 95% confidence intervals [CI], 1.51–6.28). After adjusting the models for SES, lifestyle factors, and medical history, the OR increased from 2.90 (95% CI, 1.40–6.01) to 3.43 (95% CI, 1.62–7.27). The OR for hearing loss for participants with blue-collar jobs compared with that of participants with white-collar jobs was not significant (age- and sex-adjusted OR, 1.45; 95% CI, 0.93–2.25). Older age and a history of angina pectoris or cardiovascular disease were found to increase the risk of hearing loss.

**Conclusions:**

Low educational attainment was independently associated with hearing loss in older adults without dementia in Japan.

## Background

The onset of age-related hearing loss generally occurs around 50 years of age; there are reports of people who identify themselves as having trouble hearing in one out of three individuals aged ≥65 years and more than 70% of those were aged ≥75 years [1]. The prevalence of hearing loss increases with age. Once the hearing deteriorates, it is unlikely to recover to its original condition. To date, no therapy has been identified that can fundamentally correct hearing loss, other than coping therapies, such as sensory management (e.g., the use of a hearing aid) and aural rehabilitation. Life is not immediately endangered by hearing loss; however, people with a hearing loss are excluded from communication with others, exhibit increased elderly loneliness, and experience other negative impacts on their daily lives [2]. Hearing loss can also have psychological impacts, such as social isolation and depression [3]. Although there is no evidence that hearing loss is causally related to dementia, it has been reported to be a risk factor for dementia [4, 5]. The global social cost of age-related hearing loss is 750 billion US dollars annually, which represents a substantial economic burden [6].

To prevent the development of hearing loss, it is necessary to identify its causes. Previous studies have revealed that diseases such as hypertension, dyslipidemia, and diabetes are involved in the development of hearing loss; other factors include noise, ear infections, and stress [7, 8]. More recently, social disparities have also been reported to contribute to the etiology of hearing loss [9–12]. In Europe and the United States, socioeconomic factors and hearing loss have been reported to be interrelated [13–16]. However, few studies have been conducted to explore this relationship, and it has not yet been confirmed in Japan. Low socioeconomic status (SES) is a risk factor for hearing loss in Europe and the United States, but it remains to be confirmed whether this applies in Japan. Furthermore, few studies have examined the relationship between hearing loss and socioeconomic factors after having adjusted for dementia. Therefore, to prevent hearing loss, it is necessary to evaluate the etiology of age-related hearing loss from various viewpoints, such as considering the influence of dementia.

In this study, we aimed to evaluate associations between socioeconomic factors and age-related hearing loss, with respect to dementia, in Japan. Determination of these associations may enable the prevention of age-related hearing loss at an earlier age.

## Methods

### Participants

We used the data from Toyama Dementia Survey [17, 18]. Our study included 1537 participants, all residents of Toyama Prefecture and aged ≥65 years (total population = 307,582; sampling rate = 0.5%). We chose participants from the Basic Resident Register on October 1, 2013. Public health nurses contacted the selected individuals via a phone call; those who gave consent to participate were revisited. One thousand three hundred three individuals consented to participate (acceptance rate: 84.8%). If participants were not able to respond, family members and institutional staff supported the survey. The survey items comprised participants’ demographic attributes, socioeconomic factors, and hearing loss situation. We investigated lifestyle, history, and the presence of dementia as factors related to hearing loss. Of the 1303 individuals, 129 provided incomplete responses, 135 had dementia and were excluded: thus, 1039 individuals (126 patients with hearing loss and 913 controls) were included in the final analysis. A total of 624 family members and 13 institutional staff supported the survey except for 402 individuals who were living alone. Of the 624 family members, 607 were living together.

Participation in the Toyama Dementia Study was voluntary. All participants or their family members provided written informed consent prior to participation. The Ethics Committee of the University of Toyama approved the study protocol.

### Hearing loss

Participants’ self-reported current level of hearing loss was classified as either “yes” or “no”. Those with hearing loss further classified their impairment (including the use of hearing aids) as being either inconvenient or barely able to hear.

### Demographic factors and socioeconomic status

We surveyed participants with respect to their sex, age, educational attainment, occupation, lifestyle factors, and medical history. Educational attainment was stratified as follows: ≤6 years, 7–9 years, and ≥ 10 years.

According to the Japan Standard Occupational Classification [19], occupations were classified as white-collar, blue-collar, both, or other. White-collar referred to a job in administration or management, specialist professional jobs, and clerical, sales, or service jobs. Blue-collar referred to a job in security, agriculture, forestry or fisheries, manufacturing, transport or machine operation, construction or mining, and carrying, cleaning, or packaging. “Both” referred to those who had engaged in both white- and blue-collar jobs, while “other” referred to those who had engaged in other types of employment (e.g., had been a housewife).

### Lifestyle factors

For lifestyle factors, alcohol consumption and smoking habits were surveyed. Regarding alcohol consumption, participants were categorized as current drinkers, former drinkers, or non-drinkers. Current drinkers were those who consumed alcohol daily, occasionally, or within the preceding year; former drinkers were those who had ceased alcohol consumption for ≥1 year; and non-drinkers were those who had never habitually consumed alcohol. For smoking, participants were categorized as current smokers, former smokers, or non-smokers. Current smokers were those who smoked daily or within the preceding year; former smokers were those who had stopped smoking for ≥1 year; and non-smokers were those who had never habitually smoked.

### Medical history

Participants’ medical history was assessed in accordance with International Classification of Diseases, Tenth Revision (ICD-10) criteria [20]. The conditions of interest for this study were hypertension, hyperlipidemia, diabetes, stroke, and angina pectoris/cardiovascular disease.

### Dementia diagnosis

A detailed procedure for the diagnosis of dementia has been published elsewhere [21]. In brief, a two-phase study design was used. During phase I, participants who scored < 20 on the Hasegawa Dementia Scale-Revised (HDS-R), or those who reported a history of dementia, continued into phase II of the study. Borderline participants were comprehensively judged at the time of screening and were also included in phase II. We used HDS-R which is commonly used in Japan to screen for dementia. The HDS-R has acceptable validity with a sensitivity and specificity (cutoff score = 20/21) of 0.90 and 0.82, respectively [22]. During phase II, psychiatrists visited the participants and diagnosed dementia based on the ICD-10 criteria [20].

### Statistical analysis

The chi-squared test was used to determine whether socioeconomic variables, lifestyle variables, and medical history differed between participants with and without hearing loss among those without dementia. A logistic regression analysis was used to test whether SES was associated with hearing loss; hearing loss was the dependent variable, and sex, age, lifestyle factors, and medical history were the independent variables. We created several multivariate models to inspect whether lifestyle factors, or medical history explained any observed associations between socioeconomic factors and hearing loss [23]. First, we calculated the age- and sex-adjusted odds ratios (OR) for hearing loss based on educational attainment, occupation, and socioeconomic factors. Lifestyle variables were subsequently added to the model. Next, medical history variables were added. We examined the goodness of fit of the logistic regression models using the Hosmer–Lemeshow test. We used SPSS version 23 software (IBM Corp., Armonk, NY) for all analyses. ORs and 95% confidence intervals (CIs) were calculated for the logistic regression analysis.

## Results

Table 1 summarized demographic characteristics of the participants. The proportion of participants with hearing loss significantly differed according to their age, educational attainment, occupation, and history of angina pectoris or cardiovascular disease.

**Table 1:** Presence of self-reported hearing loss and participant characteristics

**Characteristic**			**Presence of self-reported hearing loss**						***P*-value**
			**Yes (*n*** **= 126)**		**No (*n*** **= 913)**		**Total (*n*** **= 1039)**		
			***n***	**%**	***n***	**%**	***n***	**%**	
**Attribution**	Age (years)	65–74	31	24.6	521	57.1	552	53.1	< 0.001
		75–84	63	50.0	308	33.7	371	35.7	
		≥85	32	25.4	84	9.2	116	11.2	
	Sex	Male	49	38.9	404	44.2	453	43.6	0.292
		Female	77	61.1	509	55.8	586	56.4	
**Educational attainment**	Years of education	≤6 years	17	13.5	25	2.7	42	4.0	< 0.001
		7–9 years	50	39.7	338	37.0	388	37.3	
		≥10 years	59	46.8	550	60.2	609	58.6	
**Occupation**	Occupations held	White-collar	42	33.3	385	42.2	427	41.1	0.021
		Blue-collar	58	46.0	317	34.7	375	36.1	
		Both	15	11.9	159	17.4	174	16.7	
		Other	11	8.7	52	5.7	63	6.1	
**Lifestyle**	Alcohol consumption habits	Current drinker	42	33.3	327	35.8	369	35.5	0.256
		Former drinker	14	11.1	64	7.0	78	7.5	
		Non-drinker	70	55.6	522	57.2	592	57.0	
	Smoking habits	Current smoker	14	11.1	92	10.1	106	10.2	0.207
		Former smoker	26	20.6	257	28.1	283	27.2	
		Non-smoker	86	68.3	564	61.8	650	62.6	
**Medical history**	Hypertension	Yes	63	50.0	426	46.7	489	47.1	0.506
		No	63	50.0	487	53.3	550	52.9	
	Hyperlipidemia	Yes	16	12.7	172	18.8	188	18.1	0.108
		No	110	87.3	741	81.2	851	81.9	
	Diabetes	Yes	16	12.7	139	15.2	155	14.9	0.507
		No	110	87.3	774	84.8	884	85.1	
	Stroke	Yes	13	10.3	64	7.0	77	7.4	0.202
		No	113	89.7	849	93.0	962	92.6	
	Angina pectoris or cardiovascular disease	Yes	17	13.5	64	7.0	81	7.8	0.019
		No	109	86.5	849	93.0	958	92.2	

Table 2 shows the effect of education and occupation on the frequency of hearing loss, before and after adjusting for lifestyle variables and medical history. The lower the participants’ SES, the more frequently they had hearing loss. Participants with educational attainment of ≤6 years were more likely to suffer from hearing loss [age- and sex-adjusted OR 3.08 (95% CI, 1.51–6.28); model 1]. The OR for hearing loss for an educational attainment of 7–9 years did not significantly differ from that of an educational attainment ≥10 years. After adjusting the models for SES, lifestyle factors, and medical history, the OR increased to 2.90–3.43 (models 3–5). The OR for hearing loss for participants with blue-collar jobs compared with that of participants with white-collar jobs was not significant (age- and sex-adjusted OR 1.45 [95% CI, 0.93–2.25]; model 2); the OR remained stable after additional adjustment for SES, lifestyle, and medical history (models 3–5). After being adjusted for all potential confounders, a low educational attainment, older age, and a history of angina pectoris or cardiovascular disease were found to increase the risk of hearing loss (model 5).

**Table 2:** Association of self-reported hearing loss with socioeconomic status after adjusting for dementia and other factors

		**Model; odds ratio (95% confidence interval)**				
	**Crude odds**	**1**	**2**	**3**	**4**	**5**
**Educational attainment**						
≤ 6 years 7–9 years	6.34 (3.24–12.41) 1.38 (0.92–2.06)	3.08 (1.51–6.28) 1.17 (0.77–1.76)		2.90 (1.40–6.01) 1.08 (0.70–1.68)	3.06 (1.46–6.41) 1.11 (0.72–1.73)	3.43 (1.62–7.27) 1.11 (0.71–1.73)
≥ 10 years	1.00	1.00		1.00	1.00	1.00
**Occupation**						
White-collar	1.00		1.00	1.00	1.00	1.00
Blue-collar	1.68 (1.10–2.56)		1.45 (0.93–2.25)	1.35 (0.84–2.15)	1.33 (0.83–2.12)	1.27 (0.79–2.05)
Both	0.87 (0.47–1.60)		0.86 (0.46–1.62)	0.81 (0.43–1.55)	0.82 (0.43–1.56)	0.82 (0.43–1.56)
Other	1.94 (0.94–4.00)		1.51 (0.71–3.21)	1.45 (0.68–3.12)	1.34 (0.62–2.92)	1.35 (0.62–2.95)
**Age (years)**						
65–74	1.00	1.00	1.00	1.00	1.00	1.00
75–84	3.44 (2.19–5.41)	3.13 (1.98–4.96)	3.25 (2.06–5.13)	3.03 (1.91–4.80)	3.21 (2.01–5.13)	3.06 (1.90–4.93)
≥ 85	6.40 (3.71–11.04)	5.00 (2.81–8.91)	5.95 (3.43–10.34)	4.81 (2.69–8.60)	5.50 (3.02–10.02)	5.03 (2.73–9.29)
**Sex**						
Men	1.00	1.00	1.00	1.00	1.00	1.00
Women	1.25 (0.85–1.83)	1.04 (0.70–1.56)	1.10 (0.74–1.65)	1.05 (0.70–1.58)	1.03 (0.51–2.07)	1.07 (0.53–2.16)
**Alcohol consumption**						
Current drinker	0.96 (0.64–1.44)				1.61 (0.95–2.75)	1.65 (0.96–2.82)
Former drinker	1.63 (0.87–3.06)				1.79 (0.89–3.59)	1.80 (0.89–3.66)
Non-drinker	1.00				1.00	1.00
**Smoking**						
Current smoker	1.00 (0.54–1.83)				1.20 (0.53–2.71)	1.19 (0.53–2.69)
Former smoker	0.66 (0.42–1.05)				0.54 (0.27–1.11)	0.51 (0.25–1.05)
Non-smoker	1.00				1.00	1.00
**Hypertension**						
Yes	1.14 (0.79–1.66)					0.93 (0.62–1.39)
No	1.00					1.00
**Hyperlipidemia**						
Yes	0.63 (0.36–1.09)					0.60 (0.33–1.09)
No	1.00					1.00
**Diabetes**						
Yes	0.81 (0.47–1.41)					0.86 (0.48–1.53)
No	1.00					1.00
**Stroke**						
Yes	1.53 (0.82–2.86)					1.57 (0.80–3.10)
No	1.00					1.00
**Angina pectoris/cardiovascular disease**						
Yes	2.07 (1.17–3.66)					1.86 (1.00–3.44)
No	1.00					1.00

Model 1 was adjusted for age, sex, and educational attainment

Model 2 was adjusted for age, sex, and occupation

Model 3 was adjusted for age, sex, and socioeconomic status

Model 4 was adjusted for age, sex, lifestyle, and socioeconomic status

Model 5 was adjusted for age, sex, lifestyle, medical history, and socioeconomic status

## Discussion

The current study found that ≤6 years of educational attainment was associated with hearing loss, after excluding people with dementia among those aged ≥65 years and adjusting for lifestyle and medical history associated with SES.

The risk of hearing loss was approximately 3.4 times greater for participants with ≤6 years of education than for those with ≥10 years of education. This finding suggests that low educational attainment is an important risk factor for hearing loss. The risk of hearing loss for low educational attainment of ≤6 years fell after adjusting for age, sex, and work history, suggesting that age, sex, and work history are confounding factors of hearing loss for low educational attainment. On the other hand, the risk of hearing loss in low educational attainment was increased after adjusting for lifestyle and medical history, suggesting that lifestyle and medical history are protective factors for hearing loss in low educational attainment. Low SES has been reported to be associated with unfavorable habits such as smoking [24]. In Europe and the United States, several reports have suggested that low SES is associated with hearing loss [25–27]. The United Kingdom studies in which educational history (e.g., less than or more than college) and economic status (income and deprivation index) were used as an indicator of SES, reported that non-white people and people with low SES had excess risk of hearing loss [25, 27]. In the United States’ study educational history (e.g., less than or more than high school) and economic status (annual household income, poverty income ratio, insurance etc.) were used as indicators of SES, and this study reported high percentage of low SES in black and Mexican Americans, while hearing loss was less common among black population [26]. This suggested that intrinsic factors (e.g., genetics,) as well as SES were associated with hearing loss.

In the current study, blue-collar versus white-collar in terms of job history was used as an indicator of low SES. Blue collar workers are likely to be exposed to higher noise exposure than white collar workers. However, the results of the present study did not show a significant difference in the risk of hearing loss in those with blue-collar jobs when compared with those with white-collar jobs. Adjusting for age and sex, educational history, lifestyle, and medical history lowered the risk of hearing loss, suggesting that these adjustment factors are confounding factors for blue-collar hearing loss. As reported in previous studies [28–30], lifestyle factors, such as drinking and smoking, are related to hearing loss. It was suggested that the blue-collar population had undesirable lifestyle habits, such as drinking and smoking, and was more susceptible to diseases.

Among the factors adjusted for hearing loss, previous angina pectoris or myocardial infarction was strongly associated with hearing loss. It has been reported that blood flow in the inner ear and brain is impaired in the presence of lifestyle-related diseases, negatively affecting hearing function [31, 32], and that arterial stiffness is largely associated with age-related hearing loss [33], and the present results are similar to those of previous studies. It is thought that the result of arteriosclerosis, which can be called systemic aging, appeared as hearing loss, angina pectoris and myocardial infarction.

The results of this study suggest that the low SES status (low educational background) may be associated with a blue-collar work history, undesirable lifestyle habits such as drinking and smoking, and the development of lifestyle diseases, which may worsen systemic atherosclerosis, which in turn may lead to hearing loss. In the future, reducing the risk of low SES environments, in terms of disease incidence, may lead to the prevention of hearing loss.

Several limitations of this study should be noted. First, this study does not consider occupational and recreational noise exposure, which is one of the risk factors of hearing loss. However, most blue-collar occupations with high noise exposure were included in the blue-collar population, which could have been an alternative indicator. Second, the duration of hearing loss was not examined in this study and hearing tests were not conducted. The percentage of participants who reported hearing loss was less than the prevalence of hearing loss reported in the literature. In this study, hearing ability in everyday life was assessed, rather than hearing ability measured by pure tone audiometry. Previous studies have reported that more than half of individuals examined exhibited hearing loss when using pure tone audiometry, but they were unaware of this loss [34]. Therefore, there may be a stronger association between SES and hearing loss, and the prevalence of hearing loss in the present study could be an underestimate. Third, there were few participants with a medical history of ear disease associated with hearing loss, and we did not adjust for this. However, it is thought that the influence of this on the results of this study was small, because there was no relationship between a medical history of ear disease and SES. Fourth, the retrospective collection of responses could have implications for the accuracy of the data. Fifth, there is a possibility of selection bias, as we may have chosen participants who were relatively more cooperative and who led relatively healthier lifestyles. It is also possible that they selected someone with high hearing and cognitive functioning at the time of the initial telephone request for survey cooperation. However, with the exception of those who lived alone, family members may have answered the phone, and there may have been less bias because the family members attend the survey to help older adults. Sixth, there is a survival bias in the research design. However, based on the assumption that hearing loss is dangerous and the risk of death is high, if the data of individuals who died were included, the actual relevance of any associations would increase. Although there are some limitations, the novelty of this study is that we were able to accurately omit the effects of dementia and report an association between SES and hearing loss.

## Conclusions

After adjusting for lifestyle and history of medically diagnosed disease, low educational attainment (≤6 years) was independently associated with self-reported hearing loss in older adults without dementia in Japan.

## Data Availability

The datasets analyzed during the current study are available from the corresponding author on reasonable request.

## Abbreviations

SES: Socioeconomic status
CI: Confidence interval
OR: Odds ratio
ICD-10: International Classification of Diseases, Tenth Revision
HDS-R: Hasegawa Dementia Scale-Revised
